# Ectomycorrhizal Fungi Dominated the Root and Rhizosphere Microbial Communities of Two Willow Cultivars Grown for Six-Years in a Mixed-Contaminated Environment

**DOI:** 10.3390/jof8020145

**Published:** 2022-01-30

**Authors:** Maxime Fortin Faubert, Michel Labrecque, Mohamed Hijri

**Affiliations:** 1 Institut de Recherche en Biologie végétale, Département de Sciences Biologiques, Université de Montréal and Jardin Botanique de Montréal, 4101 Sherbrooke East, Montreal, QC H1X 2B2, Canada; maxime.fortin.faubert@umontreal.ca (M.F.F.); michel.labrecque@umontreal.ca (M.L.); 2 African Genome Center, Mohammed VI Polytechnic University (UM6P), Lot 660, Hay Moulay Rachid, Ben Guerir 43150, Morocco

**Keywords:** *Salix*, phytoremediation, short-rotation intensive culture (SRIC), soil contaminants, fungi, bacteria, archaea, microbiome, amplicon sequencing

## Abstract

There is a growing interest in plant microbiome’s engineering to optimize desired functions such as improved phytoremediation. This study is aimed at examining the microbial communities inhabiting the roots and rhizospheres of two *Salix miyabeana* cultivars that had been grown in a short-rotation intensive culture (SRIC) system for six years in a soil contaminated with the discharge from a petrochemical factory. DNA was extracted from roots and rhizospheric soils, and fungal ITS and bacterial and archaeal 16S rDNA regions were amplified and sequenced using Illumina MiSeq technology. Cultivars ‘SX61’ and ‘SX64’ were found to harbor a similar diversity of fungal, bacterial, and archaeal amplicon sequence variants (ASVs). As expected, a greater microbial diversity was found in the rhizosphere biotope than in the roots of both cultivars, except for cultivar ‘SX64’, where a similar fungal diversity was observed in both biotopes. However, we found that microbial community structures were cultivar- and biotope-specific. Although the implication of some identified taxa for plant adaptability and biomass production capacity remains to be explored, this study provides valuable and useful information regarding microbes that could potentially favor the implantation and phytoremediation efficiency of *Salix miyabeana* in mixed contamination sites in similar climatic environments.

## 1. Introduction

The microbiome, or to some extent, the phytomicrobiome, refers to the community of microorganisms (i.e., fungi, bacteria, and archaea) that collectively colonized most parts of the plant, including the phyllosphere, rhizosphere, and endosphere [1]. The associated microbes are so important for plant health and growth that the plant and all of its microscopic partners are no longer seen as ‘individual’, but are rather regarded as a metaorganism or holobiont [2,3].

Interactions between plants and microorganisms are known to be very ancient, because association with arbuscular mycorrhizal fungi (AMF) is believed to have played a key role in the plant terrestrialization process over 460 million years ago [4]. Other associations between plants and fungi appeared later in the evolution, and there are now several other categories of mycorrhizal symbioses (i.e., arbuscular, arbutoid, ectendo, ecto, ericoid, monotropoid, orchid, and sebacinoid) [5,6]. AMF and ectomycorrhizal fungi (EMF) have been extensively studied, because of their respective ubiquity and great diversity. Nowadays, it is estimated that AMF are still present in more than 72% of vascular land plant species and encompass a specific group of an early divergent fungal lineage belonging to the subphylum Glomeromycotina [5,6,7,8]. While EMF can associate only with a small fraction of the total number of terrestrial plants, their disproportionate occupancy of the terrestrial land greatly increased the global importance of this type of mycorrhizas [5,9]. AMF form highly branched hyphal structures (arbuscules) within cortical root cells (without penetrating the plasmalemma), while EMF envelop the root tip with a sheath or mantle and form a network of intercellular hyphae (Hartig net) between the cortical and epidermal cells [5]. The arbuscules and Hartig nets are effective interfaces to give fungi direct access to the plant’s carbohydrates. Both AMF and EMF have extraradical mycelia with a great capacity to extend beyond the root zone, which greatly benefits the growth and health of the plant, allowing it to absorb a greater amount of water and nutrients, which would not be accessible otherwise [9]. Association with mycorrhizal fungi is also known to provide plant protection against desiccation and pathogens that would enter through the roots [10,11,12]. In many plant species, the inner parts of the roots are also frequently colonized by nonmycorrhizal ascomycetes, which are collectively referred to as dark septate endophytes (DSE) [9]. Even if their presence in plant roots has been known for over a century, their function and taxonomic affinities are still elusive [13]. Studies in experimental systems have suggested that DSE could be commensalistic or mutualistic symbionts, or even latent saprotrophs or pathogens [13]. Their prevalence in harsh conditions, such as drought, nutrient-limited environments, or contaminated lands, suggests that DSE might be critical for plants health in unfavorable ecosystems [14]. Plant growth-promoting rhizobacteria (PGPR) are other beneficial microorganisms that can be found in the rhizosphere and the rhizoplane, as well as in the interior part of the roots. PGPR can directly enhance plant growth and health, in addition to offering protection to plants against phytopathogens by saturating the niche or by the induction of systemic resistance [15]. Induced systemic resistance (ISR) would also enhances plant resistance against trace elements (TEs) and organic pollutants [15]. Archaea are now considered as other important components of the plant microbiome [16,17,18]. Although less is known about their relations with plants, it is assumed that they would interact positively due to their ubiquitous occurrence within the microbiome of healthy plants [16].

In the last few years, there has been increasing interest in plant-microbiome manipulations in order to push the beneficial interactions between plants and microbes toward enhanced specific outcomes, leading to a more sustainable agriculture [19,20], or even to increased phytoremediation efficiency [3,21]. Extremely inconsistent results can be found in the scientific literature, revealing that manipulating the plant microbiome toward a certain type of community turned out to be very challenging [1]. Interactions between plants and microorganisms are very complex and not well understood. Microbial communities often vary between plant species/cultivars, and they also depend on many environmental factors, such as climatic conditions, edaphic properties, and biological interactions [1]. Facing this complexity, it is crucial to improve our knowledge concerning the natural relationships between all partners belonging to a holobiont established in a particular environmental condition before embarking on such microbiome engineering approaches.

Microbiome characterization is then a critical preliminary step to get there, and new sequencing technologies have recently allowed researchers to gain a new perspective on the microbial diversity associated with plants. Fast-growing willow shrubs (*Salix* spp.) present attractive economic values as woody crops for biomass production [22,23], and they shown interesting versatility to be used in various environmental projects, to minimize the leaching of pesticides from agricultural fields [24,25], to treat contaminated leachate [26,27], or to remediate contaminated soil [28,29,30]. Consequently, the microbial community of *Salix* spp. have been extensively studied in many different environmental conditions, such as floodplain [31], arable sites [32], and contaminated land [33,34]. However, studies concerning microbiome characterization of *Salix* spp. conducted in contaminated conditions were mostly performed on relatively young hosts [33,34,35,36,37,38,39,40], and less is known about the natural microbiome of willows established for several years on contaminated soil. The aim of this study was to determine the microbial communities found in the roots and the rhizosphere of mature willows growing under contaminated conditions. This study was then conducted inside the boundaries of a six-year-old willow plantation, containing two cultivars of *Salix miyabeana* (‘SX61’ and ‘SX64’) that had been established and cultivated under a short-rotation intensive culture (SRIC) system on a former industrial site in southern Quebec Province, Canada. Fungal, bacterial, and archaeal communities inhabiting the roots and the rhizosphere were described using an Illumina MiSeq sequencing system. We expected to observe similar microbial communities between the two cultivars, as well as finding greater microbial diversity in the rhizosphere than in the roots of both cultivars. The relevant information could provide valuable and useful clues to improve some microbiome engineering approaches favoring the establishment, survival, growth, and fitness, as well as the remediation performances, of *Salix* spp. on contaminated sites.

## 2. Materials and Methods

### 2.1. Experimental Site

This study was carried out in the municipality of Varennes (QC, Canada, 45°42′02.8″ N, 73°25′53.4″ W), located on the south shore of the St. Lawrence River, across from the Island of Montreal. The region has a climate characterized by annual average temperature of 6.6 °C and annual average precipitation of 981 mm [41]. Less than 300 m separate the centroid of the experimental site from the St. Lawrence River. This site is a flat area of 5840 m^2^, that rises approximately 3.5 m above the river water level. Between 1972 and 1979, this site was used for the purpose of land farming practices to treat settling sludge, derived from the liquid discharge of a former petrochemical factory (Pétromont Inc., Varennes, QC, Canada) that operated for many years before being shut down in 2008 [42].

In 2010, agronomic properties and contaminant concentrations were characterized in the soil (Table 1). Based on the provincial Land Protection and Rehabilitation Regulation, RLRQ, c. Q-2, r. 37, Sch. I, polychlorinated biphenyl (PCB), copper (Cu), chromium (Cr), and anthracene were considered to be the most problematic contaminants on the site. As described in Guidi et al. [43], the sector was mainly contaminated within the first 60 cm of the soil.

**Figure 1:**
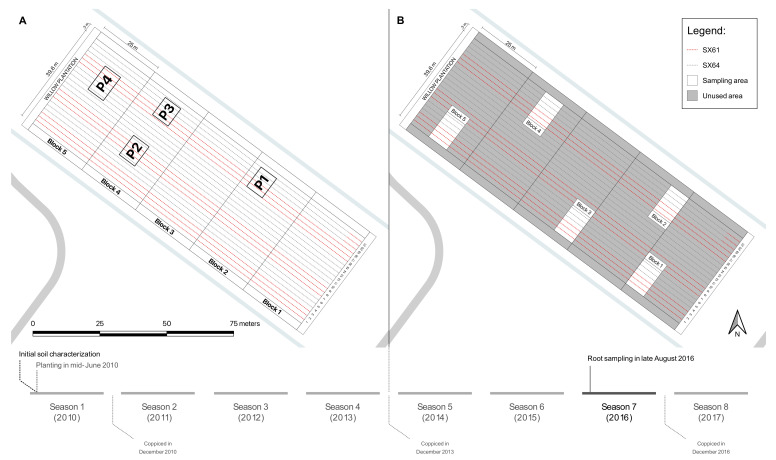
Evolution of the experimental design over time, including growth seasons and coppicing times. The 21 dotted lines inside the willow plantation refer to the rows planted with the cultivar ‘SX61’ (red lines) and with the cultivar ‘SX64’ (grey lines). (**A**) Experimental design of the first experimental phase is referred to as the GERLED site in Guidi et al. [43]. P1, P2, P3, and P4 were the sampling plots in their study; (**B**) Experimental design of the current experiment. White plots refer to the sampling areas. Although preserved as part of the plantation, the sections in dark grey were not used in the present study (Unused area). Adapted from Guidi et al. [43].

In mid-June 2010, two *Salix miyabeana* cultivars (‘SX61’ and ‘SX64’) were successfully established on part of the site (5475 m^2^) following a SRIC technique for remediation purposes [43]. The planting was carried out mechanically, and plants were spaced 1.8 × 0.3 m, at a density of 18,500 plants per hectare [43,44]. The plantation included seven randomly distributed groups of three rows, for a total of 21 rows (Figure 1A). All willows were coppiced at the end of the first growing season (December 2010) and coppiced again at the end of the fourth growing season (December 2013).

### 2.2. Sample Collection

In late August 2016, three individual plants of both cultivars (‘SX61’ and ‘SX64’) were randomly selected in each of the five blocks (*n* = 15 for each cultivar) (Figure 1B). In the field, their root systems were dug up from the soil surface (0–15 cm depth) and vigorously stirred to remove excess soil. Maximum fine roots were collected from the same tree and mixed in a 50 mL polypropylene tube (Sarstedt Inc, Newton, NC, USA) to create one composite sample by plant. In the laboratory, soil still attached to the roots was collected in 1.5 mL Eppendorf tubes (Eppendorf Canada Ltd., Mississauga, ON, Canada). This soil was considered to be part of the rhizosphere and is referred to as the rhizospheric soil sample. Composite root samples (*n* = 15 for each cultivar) and composite rhizospheric soil samples (*n* = 15 for each cultivar) were stored at −80 °C before DNA extraction processing. Root samples from each cultivar shall be referred to as Roots.SX61 and Roots.SX64, while the rhizospheric soil samples shall be referred to as Rhizo.SX61 and Rhizo.SX64.

### 2.3. DNA Extractions

Total rhizosphere genomic DNA was extracted from 0.3 g of well-mixed rhizospheric soil (wet weight) using a MO BIO’s PowerSoil DNA Isolation Kit (QIAGEN, Toronto, ON, Canada), following the manufacturer’s instructions. The roots were cleaned with tap water to remove soil particles and then crushed in liquid nitrogen using a pestle and mortar. The roots’ total DNA (including plant and endophytic DNA) was extracted from 0.1 g of well-mixed wet material using a DNeasy Plant Mini Kit (QIAGEN, Toronto, ON, Canada), following the manufacturer’s instructions. DNA concentrations were measured using a NanoDrop 2000 UV-visible spectrophotometer (Thermo Scientific, Wilmington, DE, USA). All extracts were stored at −20 °C before PCR processing.

### 2.4. PCR Amplifications and Sequencing

The first step of PCR amplifications was carried out with the overhang adapter sequences CS1 (5′-ACACTGACGACATGGTTCTACA-3′) and CS2 (5′-TACGGTAGCAGAGACTTGGTCT-3′) attached to the fungal, bacterial, and archaeal gene-specific set of primers. The forward primers were designed as (5′-CS1-Gene-specific primer-3′) and the reverse primers as (5′-CS2-Gene-specific primer-3′). The fungi-specific primers ITS1F (5′-CS1-CTTGGTCATTTAGAGGAAGTAA-3′) and 58A2R (5′-CS2-CTGCGTTCTTCATCGAT-3′) were used to amplify the ITS region of the fungal ribosomal DNA [50,51]. The bacterial V3-V4 hypervariable region of the 16S rDNA was amplified using the specific primers 515F (5′-CS1-GTGCCAGCMGCCGCGGTAA 3′) and 806R (5′-CS2-GGACTACHVGGGTATCTAAT-3′) [52,53]. The archaea-specific primers Arch516F (5′-CS1-TGYCAGCCGCCGCGGTAAHACCVGC-3′) and A806R (5′-CS2-GGACTACVSGGGTATCTAAT-3′) were used to amplify the V3-V4 region of the archaeal 16S rDNA [36,53]. PCR reactions were performed in 25 µL volumes containing 0.14 µL (0.7 unit/reaction) Taq DNA Polymerase (QIAGEN Toronto, ON, Canada) for 30 cycles (bacterial 16S) or 35 cycles (fungal ITS and archaea 16S) with annealing temperatures of 55 °C (bacterial and archaea 16S) or of 45 °C (fungal ITS). The same reaction conditions using nuclease-free water instead of extracted DNA has been used as negative control, and the success of the amplification products was checked on 2% agarose gel. Using this archaeal gene-specific set of primers (Arch516F/A806R), PCR amplification was successful for all rhizospheric soil samples, but it failed for all root samples. Dilutions were made on all extracts to reduce the possible PCR inhibitor concentrations, but no amplicons were found again.

The PCR products were shipped to Genome Quebec Innovation Center (McGill University, Montreal, QC, Canada) for library preparations and sequencing. Unique barcodes (index) and the sequence of Illumina adapters required for DNA binding to the flow cells (i5 and i7) were added to each sample in the second PCR amplification. The reaction was performed with: (0.025 unit/µL) TAQ DNA Polymerase Roche FastStart High Fidelity PCR System (Sigma-Aldrich, Oakville, ON, Canada); 10 min at 95 °C and 15 cycles of: 15 s at 95 °C, 30 s at 60 °C, and 60 s at 72 °C, followed by 3 min at 72 °C at the end. The success of barcoding for each sample was verified on 2% agarose gel. DNA was quantified with a Quant-iT™ PicoGreen^®^ dsDNA Assay Kit (Life Technologies, Burlington, ON, Canada). Libraries were then generated by pooling the same amount of each sample. The pool (or library) was cleaned up with a ratio of 0.85 of AMPure beads (Beckman Coulter Canada Inc, Montréal, QC, Canada). Libraries were then quantified using a Quant-iT™ PicoGreen^®^ dsDNA Assay Kit (Life Technologies, Burlington, ON, Canada) and a Kapa Illumina GA with Revised Primers-SYBR Fast Universal kit (Sigma-Aldrich, Oakville, ON, Canada). Average size fragment was determined using a LabChip GX (PerkinElmer^®^, Waltham, MA, USA) instrument. Before sequencing, 12% of Phix control library was spiked into the amplicon pool (loaded at a final concentration of 4.5 pM) to improve the unbalanced base composition. Sequencing was carried out using the Illumina MiSeq PE250 system (Illumina Inc, San Diego, CA, USA) with a MiSeq Reagent Kit v2 500 cycles from Illumina. Sequencing was carried out with LNA^TM^ modified custom primers (Exiqon, Woburn, MA, USA). Fungal amplicons were sequenced in a separated run than bacterial and archaeal amplicons.

### 2.5. Sequence Processing

A total of 2,993,007, 3,028,836, and 469,648 raw reads of fungi, bacteria, and archaea, respectively, were obtained from the whole dataset after sequencing runs. Theses sequencing datasets were individually processed using the “DADA2” package [52] in R V.3.5.1 [54]. All primers and low-quality sequences were first trimmed and filtered from the raw reads with the “filterAndTrim” function using a min. Q score of 6 and max. expected error of 2. Error rates were learned using the “learnErrors” function, with default parameters, for the forward and reverse reads separately. Amplicon sequences were previously dereplicated from fastq files, using the “derepFastq” function, and the exact sequences were inferred using the learned error rates to remove the sequencing errors from dereplicated reads, using the “dada” function. Forward and reverse reads were then merged using the “mergePairs” function, without allowing mismatches in the overlap region. The smallest overlap regions were 12 bps in the fungal dataset, 24 bps in the bacterial dataset, and 21 bps in the archaeal dataset. Chimeras were removed with the “removeBimeraDenovo” function. Singletons, doubletons, and reads with prevalence <2 were removed from the datasets as they were considered to be artefacts. Using the “assignTaxonomy” function, taxonomic assignment of fungi was carried out using the General Fasta release files version 7.2 from the UNITE ITS as reference database [55]. The Silva training set version 128 was used for bacterial and archaeal Taxonomic assignment [56]. Archaea were also submitted to another taxonomic assignment using the RDP database [57]. Species-level annotation were then added to the bacterial and archaeal taxonomic table, using the “addSpecies” function. After filtering, a total of 1,492,944, 1,472,659, and 214,421 high-quality sequences of fungi, bacteria, and archaea, respectively, were recovered from the whole dataset (Figure S1). The taxonomic assignations were visualized with the Krona tool [58] through the “plot_krona” function in the “psadd” package [59].

### 2.6. Statistical Analysis

The rarefaction curves were calculated and visualized using the “ggrare” function in the “ranacapa” package [60], and Good’s Coverage was calculated using the “goods” function in the “QsRutils” package [61]. All rarefaction curves tended towards their horizontal asymptotes (Figure S2), and Good’s coverage estimations revealed that between 98.6–99.9%, 92.6–98.2%, and 99.5–100.0% of fungal, bacterial, and archaeal amplicon sequence variants (ASV) were obtained from their respective dataset, suggesting that the sequencing was adequate. Alpha diversity was assessed with the Shannon index using the “estimate_richness” function in the “Phyloseq” package [62]. Group means of the Shannon index were then submitted to a two-way analysis of variance (ANOVA) test, using JMP^®^ Pro V.15.0.0 (SAS Institute, Cary, NC, USA). As recommended in McMurdie and Holmes [63], a variance stabilizing transformation (VST) has been used to normalized the ASVs count data using the “varianceStabilizingTransformation” function in the “DESeq2” package [64]. VST count data was then submitted to a Permutational Analysis of Variance (PERMANOVA) using the “adonis2” function in the “vegan” package [65], as well as to Permutational multivariate Analyses of Dispersion (PERMDISP) using the “betadisper” function in the “vegan” package. PERMANOVA and PERMDISP were both based on the Euclidean distance matrix calculated using the “vegdist” function in the “vegan” package. Euclidean distances among the samples were visualized with principal component analysis (PCA). As suggested in McMurdie and Holmes [63], differential abundance analysis based on the Negative Binomial Wald test has been used with the “DESeq” function in the “DESeq2” package [64] to identify ASVs that were differentially abundant across the two sample groups. Because our PCA plots revealed that some groups had much higher within-group variability than the others, it was more appropriate to create smaller datasets before running the “DESeq2” function on our samples [64]. The root samples where then separately analyzed from the rhizospheric soil samples, and all samples from ‘SX61’ where separately analyzed from those of ‘SX64’. Creating smaller datasets with only two groups without the others is supposed to be more sensitive than a model including all samples together [64]. In this case, “baseMean counts” is the average of the normalized count values, calculated over all samples of a subset datasets (*n* = 30 for each cultivar and *n* = 30 for each compartment), rather than over the entire dataset (*n* = 60). Normalization was performed in the “DESeq2” package by dividing raw values by size factors [64]. ASVs that were differentially abundant across two sample groups were then visualized on MA-plots (minus over average-plots) using the “ggmaplot” function in the “ggpubr” package [66]. The common core microbiome was investigated at the ASV level and is defined here as the set of ASVs with >0.1% of non-transformed reads abundance in at least 14 of the 15 samples in every groups. Venn diagrams were created using the “venn” function in the “venn” package [67] to visualized the shared ASVs between the four groups.

## 3. Results

### 3.1. Fungal Community Structure

**Figure 2:**
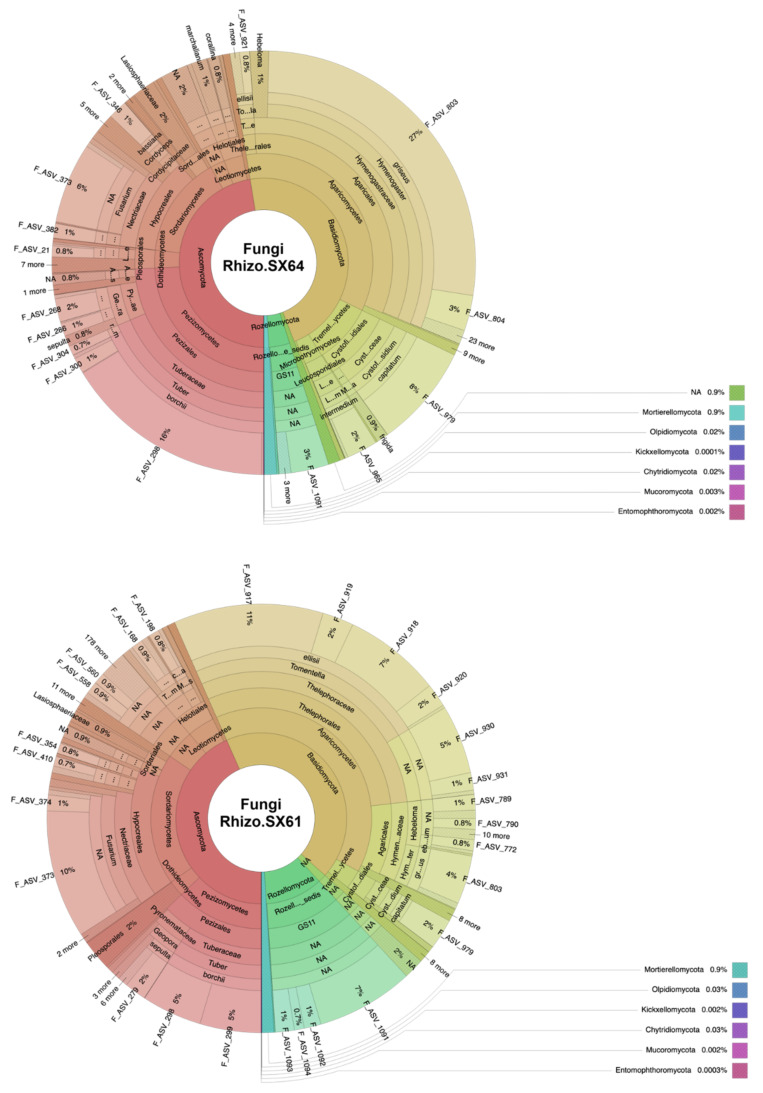

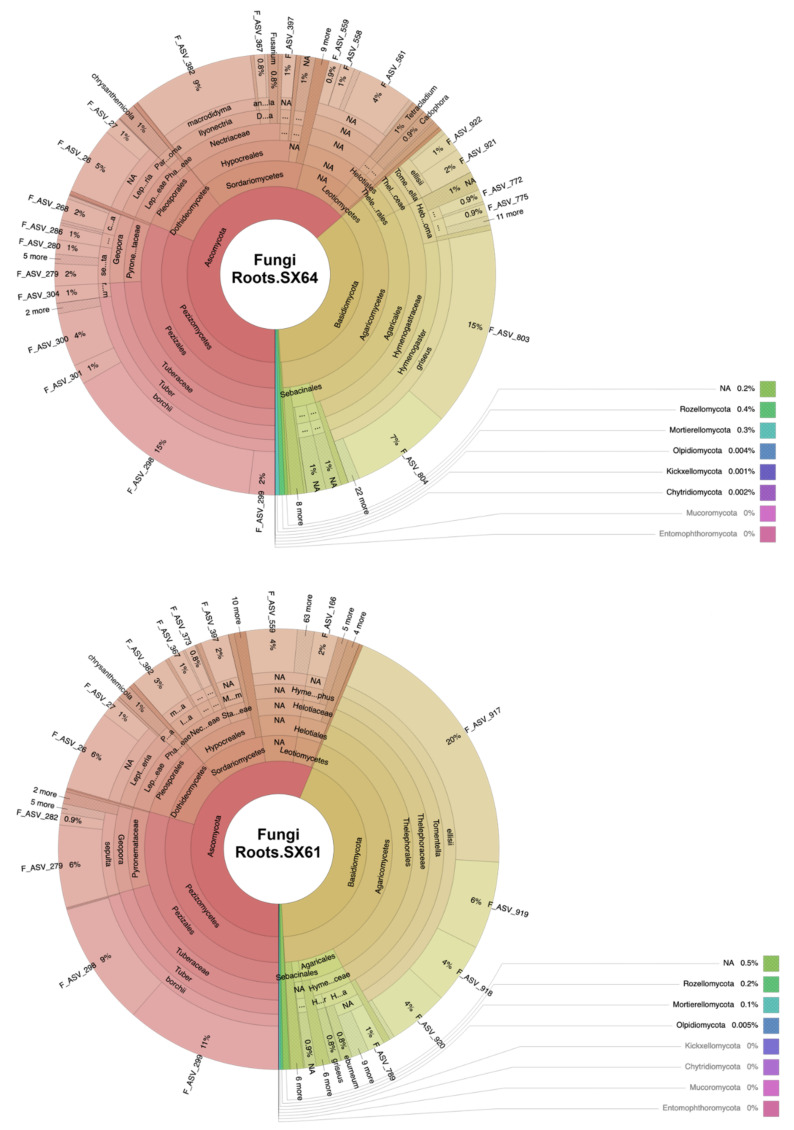
Krona charts of raw read counts of all fungal ASVs in each biotope of both *Salix* cultivars. Arc lengths are proportional to the relative number of reads by group (Rhizo.SX64 = 684,598 reads; Rhizo.SX61 = 596,370 reads; Roots.SX64 = 95,896 reads; and Roots.SX61 = 116,080 reads). The interactive Krona charts are available at https://github.com/MaximeFortinFaubert/Figure2/blob/main/README.md (accessed on 1 December 2021).

Over the 1,492,944 high-quality sequences recovered from the fungal dataset, 1292 ASVs were observed and divided into 9 phyla and 27 classes (Figure 2). One hundred ninety of these ASVs (containing 16,223 high-quality sequences, representing ~1.09% of the total fungal dataset) remained unclassified at the phylum level. For the root samples, 748 of those ASVs were observed among the two willow cultivars. Among the seven observed phyla in the root samples, Basidiomycota and Ascomycota were the most dominant taxa, representing 43% (49,889 reads) and 56% (65,282 reads) of the sequences in ‘SX61’ and 35% (33,988 reads) and 64% (61,007 reads) in ‘SX64’. The abundances of other phyla such as Chytridiomycota, Kickxellomycota, Mortierellomycota, Olpidiomycota, and Rozellomycota were less than 1%. In both cultivars, Pezizomycetes, Dothideomycetes, Sordariomycetes, and Leotiomycetes were the most predominant classes of Ascomycota. Agaricomycetes was the only dominant class of Basidiomycota phylum in both cultivars, accounting for 43% (49,797 reads) of the fungal community in ‘SX61’ and 35% (33,815 reads) in ‘SX64’. In the rhizosphere samples, a total of 1288 ASVs were assigned into 9 phyla and 27 classes, which were observed in the two cultivars. In both, Basidiomycota, Ascomycota, and Rozellomycota were the three most dominant phyla. Basidiomycota represented 43% (257,828 reads) of the sequences in ‘SX61’ and 47% (319,653 reads) in ‘SX64’. Ascomycota also represented 43% (258,657 reads) of the sequences in ‘SX61’ and 47% (324,732 reads) in ‘SX64’. Rozellomycota represented 11% (65,022 reads) of the sequences in ‘SX61’ and 4% (27,242 reads) in ‘SX64’. The abundances of other phyla such as Chytridiomycota, Entomophthoromycota, Kickxellomycota, Mortierellomycota, Mucoromycota, and Olpidiomycota were less than 1%. At the class level, Agaricomycetes, Tremellomycetes, and Microbotryomycetes were the three most dominant classes of the Basidiomycota phylum, with an abundances of 40% (235,932 reads), 3% (16,626 reads), and 0.5% (3073 reads) in ‘SX61’ and 35% (237,817 reads), 9% (61,159 reads), and 3% (18,219 reads) in ‘SX64’, respectively. Within Ascomycota, Sordariomycetes, Pezizomycetes, Leotiomycetes, and Dothideomycetes were the four most dominant classes, accounting for 19% (115,238 reads), 13% (76,739 reads), 4% (23,882 reads), and 2% (13,866 reads) of the ‘SX61’ rhizospheric community, and for 15% (103,299 reads), 24% (165,793 reads), 3% (19,133 reads), and 3% (18,106 reads) of the ‘SX64’ rhizospheric community, respectively.

### 3.2. Bacterial Community Structure

The 1,472,659 bacterial sequences remaining from the whole dataset after sequence processing were clustered into 6079 ASVs (Figure S3). Only 83 of these ASVs (containing 1449 high-quality sequences, representing ~0.10% of the total bacterial dataset) remained unclassified at the phylum level. A total of 5535 of these ASVS were present among the root samples of both cultivars. The bacterial root community compositions of ‘SX61’ and ‘SX64’ were primarily dominated by the Proteobacteria (64% (175,327 reads) and 63% (172,105 reads)), followed by Actinobacteria (15% (40,266 reads) and 14% (37,770 reads)), Bacteroidetes (11% (29,446 reads) and 11% (30,220 reads)), Cyanobacteria (2% (5040 reads) and 2% (5395 reads)), and Acidobacteria (3% (9444 reads) and 4% 10,970 reads). Most of the Proteobacteria ASVs belonged to the Alphaproteobacteria, Gammaproteobacteria, Betaproteobacteria, and Deltaproteobacteria. In the rhizosphere samples, the bacterial community compositions of both cultivars (‘SX61’ and ‘SX64’) included 6020 ASVs and were also dominated by the Proteobacteria (48% (216,953 reads) and 48% (232,313 reads)), followed by Actinobacteria (21% (91,961 reads) and 17% (81,983 reads)), Bacteroidetes (10% (44,961 reads) and 12% (56,944 reads)), and Acidobacteria (8% (37,380 reads) and 9% (41,579 reads)). Other phyla such as Verrucomicrobia (2% (9270 reads) and 2% (11,926 reads)), Chloroflexi (3% (15,498 reads) and 3% (16,232 reads)), Gemmatimonadetes (3% (13,646 reads) and 3% (15,960 reads)), and Planctomycetes (3% (12,155 reads) and 3% (15,687 reads)) were present in ‘SX61’ and ‘SX64’, respectively. As in the root samples, most of the ASVs associated with Proteobacteria also belonged to Alphaproteobacteria, Gammaproteobacteria, Betaproteobacteria, and Deltaproteobacteria. Cyanobacteria were also present in the rhizosphere of both Salix, but only in less than 1% abundance.

### 3.3. Archaeal Community Structure

In the rhizospheric dataset, 34 archaeal ASVs were recovered from the 214,421 high-quality sequences and grouped into 2 phyla and 3 classes (Figure S4). Only one ASV (containing 10 high-quality sequences, representing ~0.005% of the total archaeal dataset) remained unclassified at the phylum level. Thaumarchaeota was the dominant phylum in both cultivars, with almost 100% of the total read abundance (100,460 reads in ‘SX61’ and 113,845 reads in ‘SX64’). In both cultivars, all Thaumarchaeota ASVs were identified as member of the soil Crenarchaeotic Group (SCG), according to the Silva training set. Another taxonomic assignment, using the RDP database, suggested that these ASVs were all *Candidatus Nitrososphaera*. Euryarchaeota was the only other archaeal phylum observed in the dataset, with four ASVs representing a total abundance <0.1% in both cultivars (15 reads in ‘SX61’ and 91 reads in ‘SX64’). These four ASVs were assigned to the genus level *Methanosarcina*, *Methanocella*, and *Methanobacterium* according to both databases (Silva and RDP). Seven reads of *Methanocella* sp. were found in the Rhizo.SX64 samples only, representing 8% of the Euryarchaeota reads.

### 3.4. Alpha Diversity

The bacterial diversity index (Shannon index) was significantly higher than the fungal diversity index, which were both significantly more diverse than the archaeal diversity index (*p* < 0.001 in every group sample). Table 2 shows that respective fungal, bacterial, and archaeal diversity indexes were similarly diverse between both cultivars. Greater bacterial diversity was found in the rhizosphere than in the roots of both cultivars, as expected. The fungal communities of ‘SX61’ were more diverse in the rhizosphere than in the roots, while those of ‘SX64’ did not show any significant differences between the two biotopes.

### 3.5. Beta Diversity

PERMANOVA analysis based on the Euclidean distance matrix showed that fungal and bacterial community compositions were significantly influenced by plant compartment and by cultivar (Table 3). Archaeal community composition from the rhizosphere was also strongly influenced by cultivar. PERMDIPSD analysis showed that the dispersion of the fungal communities was significantly different between both biotopes in each cultivar and that the dispersion of the archaeal communities was significantly different between both cultivars in the rhizospheric soil samples (Table S1 and Figure S5). These results suggest that the differences detected by PERMANOVA could be artifacts of heterogeneous dispersion.

**Figure 3:**
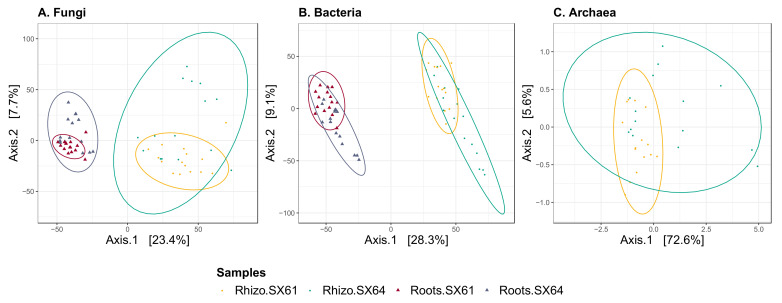
Principal component analysis (PCA) ordinations of microbial communities. Euclidean distances were calculated on the variance stabilizing transformed (VST) ASV counts in each: (**A**) fugal, (**B**) bacterial, and (**C**) archaeal datasets. Shapes (triangle and circle) represent the compartments and colors (red, blue, yellow, and turquoise) represent samples groups. Samples closer together contain more homogeneous communities than samples farther apart. Ellipses were drawn around communities based on a 95% confidence interval.

The PCA reflects those differences as fungal and bacterial roots samples showed separated cluster from the rhizospheric soil samples along the first canonical axis, which represent 23.4% and 28.3% of their total variability (Figure 3A,B). Samples also showed separation between both cultivars along the second axis, which represent an additional 7.7% and 9.1% of their total variance, respectively. PCA of archaeal communities showed that the rhizospheric soil samples from ‘SX64’ (Rhizo.SX64) were less clustered than those from ‘SX61’ (Rhizo.SX61), which typically clustered together along the first canonical axis, representing 72.6% of the total variability (Figure 3C).

### 3.6. Differential Abundance of ASVs

Many ASVs were found to have significant differential abundances (adjusted *p*-value < 0.05) between the respective biotopes of both cultivars (Rhizo.SX61 vs. Rhizo.SX64 and Roots.SX61 vs. Roots.SX64) and between biotopes of the same cultivar (Rhizo.SX61 vs. Roots.SX61 and Rhizo.SX64 vs. Roots.SX64). A total of eight fungal ASVs were significantly more abundant in the roots than in the rhizospheric soil samples of, either one or both, willow cultivars. All these ASVs belonged to Dothideomycetes (*Leptosphaeria* spp.), Leotiomycetes (*Cadophora luteo-olivacea*, *Cadophora orchidicola*), and Sordariomycetes (*Dactylonectria anthuriicola*, *Ilyonectria macrodidyma*, *Myrothecium* spp.). The only two fungal ASVs showing significant differential abundances between the root samples of both cultivars were assigned to *Hymenogaster griseus* and *Tomentella ellisii*. All differential abundances can be visualized in the MA-plots of Figure S6. A visual summary of the most abundant ASVs (with a base mean >10) with significant differential abundances between cultivars are presented in Figure S7.

### 3.7. Common Core Microbiome

**Figure 4:**
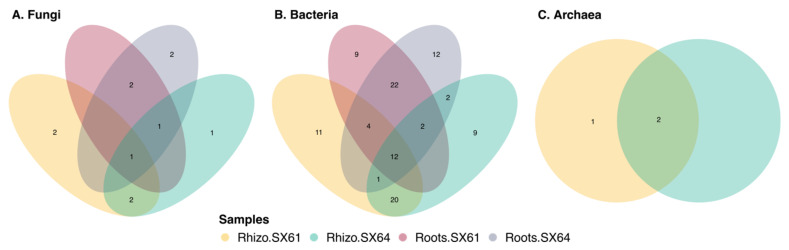
Venn diagram of shared (**A**) fungal, (**B**) bacterial, and (**C**) archaeal ASVs between all group samples.

The common core microbiome can be defined as a set of microbial taxa that occurs within a host population above a particular occupancy frequency threshold [68]. It can refer to various taxonomic ranks and have various dimensions (i.e., time and space), as well as different levels of complexity, such as plant biotope, plant population, and their phylogeny [2,69]. Here, the microbial communities were further investigated to identify the most widespread microbial taxa within the biotopes of both cultivars. As shown in Figure 4, only one fungal and 12 bacterial ASVs were considered as common to the four libraries. The unique fungal ASV was identified as *Fusarium* sp., while the bacterial ones were identified as members of Actinobacteria (*Lysinimonas* sp.), Bacteroidetes (*Terrimonas* sp.), and Proteobacteria, including five Alphaproteobacteria, one Betaproteobacteria, and four Gammaproteobacteria. The root compartment of both cultivars shared three other fungal ASVs, identified as *Leptosphaeria* sp., *Dactylonectria anthuriicola*, and *Ilyonectria macrodidyma*, as well as 28 others bacterial ASVs, identified as members of Acidobacteria, Actinobacteria, Bacteroidetes, Cyanobacteria, and Proteobacteria. Two other fungal ASVs, identified as *Tetracladium marchalianum* and *Cystofilobasidium capitatum*, as well as 21 others bacterial ASVs, identified as members of Acidobacteria, Actinobacteria, Bacteroidetes, Gemmatimonadetes, and Proteobacteria, and two archaeal ASVs, identified as part of the Nitrososphaera family, were also common in the rhizosphere of both cultivars.

## 4. Discussion

### 4.1. Beta and Alpha Diversities

In this study, we examined the root and the rhizosphere microbiomes of two cultivars of *Salix miyabeana* (‘SX61’ and ‘SX64’) that were grown under SRIC for six years in a mixed-contaminated soil. Even if new sequencing techniques allow us to improve the knowledge of plant microbiome, our results must be interpreted with caution as they were based on sampling during a single time point (in late August 2016). Multivariate analyzes revealed that microbial taxonomic compositions significantly varied between cultivars, as well as between plant biotopes. It is well known that plant identity is a strong driver of the abundance and structure of soil microbial communities [34]. Plant species and cultivars can differently influence their microbiome in the roots, in the rhizospheres, and in the adjacent bulk soils, through different exudation patterns of carbon compounds, in order to attract or feed preferred partners, or even to deter unwanted pathogens and competitors [1,40]. The PCA also showed that microbial communities in the root samples were more clustered than in the rhizospheric soil samples. This could reflect the well-known selective gradient that exists from the rhizosphere to the endosphere biotope [69]. Despite the clear separations, the first and second axis of the PCA for fungi and bacteria accounted for a small proportion of the variations observed, indicating that the relative abundance of many ASVs were quite similar between the four sample groups.

The microbial diversity is also known to decline sequentially from the rhizosphere to the interior biotope of the roots, due to an increase in competition between microorganisms as the habitat become more tightly defined [37,69]. It was then not surprising to find a significantly lower diversity of bacterial ASVs in the roots of both cultivars than in their rhizosphere. The Archaeal diversity would also have been lower in the roots than in the rhizosphere of both cultivars, because 34 ASVs were identified from the rhizospheric soil samples, while none were obtained from the root samples. Our results surprisingly showed that only cultivar ‘SX61’ harbored a significantly lower diversity of fungal ASVs in the roots than in the rhizosphere, while cultivar ‘SX64’ seemed to harbor a similar diversity between the two biotopes. Iffis et al. [70] also reported similar fungal diversity between the roots and the rhizosphere of other plant species (*Solidago canadensis*, *Populus balsamifera*, and *Lycopus europaeus*) that were grown in petroleum hydrocarbon polluted sedimentation basins.

Although the community compositions of each kingdom were found to be significantly different between both cultivars in each biotope, their alpha diversity remained statistically similar, which could reflect the differential abundances of some taxa. Increasing microbial diversity in roots and rhizosphere often leads to greater functional trait diversity, redundancy and complementarity, which would improve ecological services, as well as plant resistance to environmental changes and pathogen invasion [1,71,72]. In a contaminated environment, higher diversification of the soil microbiome have been found to be more effective for the biodegradation of some crude oil fractions [73]. Based on the similar alpha diversity between both willow cultivars, our results suggest a similar interest for their use in phytoremediation.

### 4.2. Mycorrhizal Fungi

In addition to diversity, the presence and abundance of some taxa could be relevant for the survival, growth, and phytoremediation efficiency of some plant species. It is well known that *Salix* spp. can form mycorrhizal symbiosis with both AMF and EMF [38,74,75]. Interestingly, in the present study, no ASV were identified as member of Glomeromycotina. The age of our willow shrubs could partially explain our results, because the associations between *Salix* spp. and AMF seem to be more important during early stages of growth and are later largely replaced by EMF [76,77]. Such succession could explain why Pray [78] did not find any sequence of Glomeromycotina in the roots of two-year-old *S. miyabeana* (‘SX61’ and ‘SX64’), even though they were inoculated with AMF at the establishment stage.

To validate the absence of AMF in our samples, it would have been appropriate to use 18S rDNA AMF-specific primers (such as AML1/AML2) in addition to the ITS universal fungal primers [79,80]. Moreover, simple staining of the roots, followed by microscopic examinations, could have been carried out to further validate our observations [33]. Despite the worse specificity of ITS primers for the detection of *Glomeromycotina* [79], some researchers still have successfully identified a great diversity of AMF in their samples by targeting ITS region [70,81]. Iffis et al. [82] even reported a comparable taxonomic profile of AMF, in terms of abundance, between their ITS dataset and their specific AMF one, generated from the same samples. The use of the same primers as ours did not allow Yergeau et al. [36] to identify AMF in the rhizosphere of *S. purpurea*, but it did allow Bell et al. [38] to identify some in the rhizosphere of *S. purpurea* ‘Fish Creek’, *S. miyabeana* ‘SX67’, and *S. dasyclados* ‘SV1’, even if they were all predominantly associated with EMF. Tardif et al. [37] also reported that Basidiomycota and Ascomycota occupied a greater relative abundance than Glomeromycotina in the roots and the rhizosphere of *S. purpurea* ‘Fish Creek’ and *S. miyabeana* ‘SX67’.

In the present study, Ascomycota and Basidiomycota were also the two most dominant fungal phyla in the roots, as well as in the rhizosphere of both willow cultivars. Among these two phyla, several putative EMF taxa were assigned to the ASVs observed in both cultivars, suggesting that every single plant of the plantation could have been colonized by multiple EMF, which in turn could have interacted with several other plants of both cultivars to form a common mycorrhizal network (CMN) on the site [83]. EMF partners of both cultivars were essentially taxa belonging to Pezizales (*Tuber* spp. and *Geopora* spp.), Agaricales (*Hymenogaster griseus, Hebeloma* spp.), and Thelephorales (*Tomentella ellisii*).

A strong link has been found between soil contamination and the association of *Salix* spp. with members of Pezizomycotina [37,40]. It has been suggested that pezizomycetes EMF could offer a more beneficial cost–benefit ratio to their hosts than Basidiomycota, because they would be less robust [84]. The stout hyphae and thick-walled chlamydospores and ascospores of pezizalean may further contribute to their ability to persist under disturbed environmental conditions [85]. In our samples, the two most dominant Pezizales genera were *Tuber* spp. and *Geopora* spp., which have already been reported as being among the most dominant EMF in other *Salix* spp. [32,86]. *Tuber borchii* caught our attention because it appeared to be one of the most important symbiotic partners of both willow cultivars. This hypogeous ascomycetous species has been suggested to be potentially involved in phytoremediation, due to the presence of metallothioneins in its ascocarps, which may play a critical role in the metal tolerance of both partners [87]. It also harbors numerous genes that encode for proteins, which are already known to be involved in metal tolerance pathways in the yeast *Saccharomyces cerevisieae* [88]. To our knowledge, *Tuber* spp. have never been reported to be associated with willows growing in a contaminated soil from Quebec, Canada, even if they are well known to form ectomycorrhizas with many *Salix* spp. [32,76,86,89]. Apparently, the soil water regime would be a very important factor determining the microenvironment of *Tuber* spp., and most would be very common to lowland ecosystems with a shallow water table [76,89,90]. The edaphic conditions of the experimental site, with its proximity and its low elevation from the south shores of the St-Lawrence River, could have favored the symbiotic association of *Tuber* spp. with our *Salix miyabeana* cultivars. It has been suggested that frequent harvests (shorter than 6-year rotation) of *S. viminalis* would promote its mycorrhization with *Tuber* spp. [86]. Therefore, the 3-year rotation length in our SRIC of willow could have played a key role in the selective promotion of *Tuber* spp. *Geopora* spp. appeared to be another important pezizomycete EMF partners of both *S. miyabeana* cultivars. This genus seems to be commonly dominant in willows that were grown in harsh conditions, such as fly-ash landfills [91], former landfill [38], or fields containing high concentrations of petroleum hydrocarbons [37]. *Geopora* spp. were also reported to have saprotrophic abilities [92], which is relevant to consider in phytoremediation.

*Hymenogaster griseus* and *Tomentella ellisii*, respectively belonging to the Agaricales and the Thelephorales orders, were among the most dominant basidiomycetous EMF in our samples. Even if hosted by both cultivars, our results showed that *T. ellisii* was mostly associated with ‘SX61’, while *H. griseus* was more dominant in ‘SX64’. Such differences might suggest that both cultivars exert different selective pressure for their partners, or that both fungi would not cohabit very well and would compete to inhabit the same ecological niche. In marginal farm land in Québec, *H. griseus* has been assigned to one of the 16 OTUs observed in the roots of the same two cultivars (‘SX61’ and ‘SX64’), after one season of growth [80]. In central Sweden, *H. griseus* was also identified as one of the most dominant taxa associated with four-year-old *Salix* spp. [32]. Interestingly, in the same study, 11-year-old *Salix* spp. also harbored *H. griseus*, but in a marginal proportion compared to *Tomentella* spp., which were not observed in the youngest plantations. Even if *Tomentella* spp. would not coexist very well with *Hymenogaster* spp., it seems to be commonly found together with Pezizales (*Tuber* spp. and *Geopora* spp.) in *Salix* spp., *Populus* spp., and *Quercus* spp. [32,86,90,93], suggesting that these fungi do not have strong antagonistic effects against each other.

*Hebeloma* spp. appeared to be another putative EMF partners of both cultivars. Even if these Basidiomycota are generally described as early-stage mycorrhizal symbiont [9,94,95], some have been reported in older stands of willows grown in harsh environments [76,96,97]. *Hebeloma* spp. could have important roles in contaminated soils because some have been found to harbor genes that encode for proteins already known to be involved in metal tolerance pathways in the yeast *Saccharomyces cerevisieae* [88]. *Hebeloma* spp. are among the few EMF species to be commercially available as inoculum for agricultural applications [80,98], and to be studied for phytoremediation applications [99,100].

### 4.3. Nonmycorrhizal Endophytic Fungi

Our results indicate that the healthy roots of both cultivars hosted a considerable abundance of fungi. Because roots were only washed with tap water, it is impossible to conclude that all the identified taxa were colonizing the inner parts of tissues. Root surface sterilization could have been carried out to kill what lies outside the roots, but the efficiency of this step is controversial due to a possible detection of DNA from dead cells that could reside on the rhizoplane after treatment [101].

A total of eight fungal ASVs were significantly more abundant in the roots than in the rhizospheric soil samples, of either one or both cultivars. Even if observed in the rhizospheric soil samples, their significantly higher abundance in the root samples suggest that these taxa could be endophytic fungi. These ASVs were identified as *Leptosphaeria* spp., *Cadophora luteo-olivacea*, *Cadophora orchidicola*, *Dactylonectria anthuriicola*, *Ilyonectria macrodidyma*, and *Myrothecium* spp., which belong to three classes (i.e., Dothideomycetes, Leotiomycetes, and Sordariomycetes) that appear to be commonly found in the roots of *Populus* spp. and *Salix* spp. growing in a contaminated environment in Quebec (Canada) [37,40,70,81].

The ecological function of the nonmycorrhizal fungi that colonize root tissues is sometimes difficult to determine [13], but the fact that all of them were identified from healthy roots suggests that they were not harmful for our *Salix miyabeana*. Endophytic fungi and their interactions with plants show great potential for increasing plant growth and improving phytoremediation efficiency [102]. Some taxa identified here could then have beneficial function for *Salix miyabeana* growing under contaminated conditions. For example, a *Leptosphaeria* sp. has been described as dark septate endophytic (DSE) with beneficial effects on the growth of *Ammopiptanthus mongolicus* under drought conditions [103]. Under axenic conditions, a *Cadophora* sp. has shown beneficial effects on the growth of the cuttings of *Salix caprea* in soil enriched with Cd and Zn [104].

The use of such fungi as inoculum to increase the phytoremediation potential of willows looks like an interesting avenue to explore, but it should be approached with caution. Some *Leptosphaeria* spp. are well known to contains plant pathogenic species responsible for lesions on the leaves of many cruciferous species, and they invade their stem and cause severe cankers at their root necks and stem bases [105]. *Cadophora* also includes species that are frequently encountered as postharvest fruit pathogens [106,107]. Ilyonectria includes pathogenic species often reported as causative agents of root-rot disease and rusty symptoms in ginseng and olive trees [108,109]. To our knowledge, these taxa have never been described as specific pathogens to *Salix* spp. However, as mentioned by Corredor et al. [110], some of them may act opportunistically and use the newly planted cuttings as temporal hosts, thus limiting the establishment of willow. *Cadophora luteo-olivacea*, *Ilyonectria* spp., and *Leptosphaeria sp.* can also take advantage of the death tissues of willows to colonize the tree [111]. In addition, *Cadophora* spp. can be considered as a potential threat to the health of *Salix* spp. during long cold-storage of cuttings [112].

Several endophytic fungi are opportunistic and have the ability to occupy a variety of ecological niches [113], which would explain why many taxa were observed in both biotopes. Some nonmycorrhizal taxa were quite redundant in a relatively high abundance in our root samples. Interestingly, the only one fungal ASV identified as part of the core microbiome in all group samples was assigned to a *Fusarium* sp. and was significantly more abundant in the rhizosphere of both cultivars. *Fusarium* is found to be among the most common and abundant genus in the roots of various plant species [114,115,116], including *Salix* spp. grown under SRIC [110]. This genus includes species that are commonly considered to be plant pathogens [117,118]. However, nonpathogenic *Fusarium* spp. are gaining interest in agriculture as a biocontrol agent to manage plant diseases [119].

Healthy willows hosted a considerable abundance of nonmycorrhizal endophytic fungi, which are possible opportunistic or obligate endophytes or biotrophic pathogen taxa. More studies are needed to fully understand the interactions of these taxa with willows and to assess their potential use in phytoremediation.

### 4.4. Archaeal Communities

The high dominance of Thaumarchaeota and the negligible presence of Euryarchaeota suggests that rhizospheric archaeal communities are involved in nutrient cycling, mostly as ammonia oxidizers. To our knowledge, very few studies have focused on the archaeal communities inhabiting the roots of *Salix* spp. Among them, Yergeau et al. [36] reported higher archaeal diversities in the rhizosphere of 100-day-old *Salix purpurea* that were grown in petroleum-contaminated soil, with a high dominance of both Euryarchaeota and Thaumarchaeota. Our results were in agreement with previous studies that also reported a high dominance of Thaumarchaeota in the rhizosphere of many other plant species [120,121,122,123,124,125]. Due to their preferences for anoxic conditions, Euryarchaeota appear to be mostly dominant in the rhizosphere of crops that are typically cultivated in flooded soil, such as *Oryza sativa* [126].

Compared to fungi and bacteria, little is known regarding the ecological roles of archaea inhabiting plant microbiomes, because most of them remain undetectable when using conventional culture-based methods. In the last few years, next-generation sequencing methods, as well as omics (i.e., metabolomics, metatranscriptomics, and metagenomics), have greatly increased the number of studies assessing archaeal communities, as well as their roles in plant proximity [17]. Consequently, the archaeal community is now considered as an important component of the plant rhizosphere microbiome [16,17,18]. Although the relationships between archaea and plants remain largely unclear, their ubiquitous occurrence on healthy plants has been suggested to reflect positive interactions between both partners [16].

It is expected that ammonia-oxidizing archaea (AOA) and methanogens archaeon play important roles in nutrient cycling within the rhizosphere [17]. Thereby, AOA could fulfil key functions in the context of soil bioremediation because nutrients, such as nitrogen, are often limited in petroleum-contaminated soil due to an unbalanced C–N ratio [35]. Based on metagenomic mining, it has been deduced that the archaeal community inhabiting the rhizosphere could potentially fulfill other important functions involved in plant growth promotion, such as through auxin biosynthesis, nutrient supply, and protection against abiotic stress [16].

In our study, no archaeal sequence was obtained from all root samples of both cultivars. Following the roots’ DNA extractions, serial dilutions were performed to reduce the possible PCR inhibitor concentrations that can be found in plant material, such as polysaccharides and humic acids [127]. Bovine serum albumin (BSA) has also been used as an additive to increase the yield of PCR, but no amplicons were found. Too much PCR inhibitor concentration, combined with a very low archaeal DNA concentration, may have caused the failure of generating archaeal amplicons from all our root samples. However, our results could also basically reveal that no archaea harbored inside the roots of our two willows. To our knowledge, endophytic archaea have never been reported from the roots of *Salix* spp. However, a few studies conducted on other plant species reported internal colonization by archaea, as in cherries of *Coffea arabica* [128]; in roots of *Zea mays* [101]; in leaves, stems, and roots of *Phragmites australis* [129]; in roots of *Oryza sativa* [130]; in leaves of *Olea europaea* [131]; and in roots of *Solanum* sp. [123]. Nevertheless, as mentioned by Chelius and Triplett [101], such results can be questionable, considering that some taxa may have been incorrectly classified as endophytes due to a possible detection of DNA from dead cells that reside outside after surface sterilization.

## 5. Conclusions

According to the presented results in this study, it could be concluded that the diversity of the fungal, bacterial, and archaeal community was quite similar between both cultivars (‘SX61’ and ‘SX64’) of six-years-old *Salix miyabeana* that grown in a mixed contaminated soil from the southeast region of Canada. Microbial diversity generally decreased from the rhizosphere to the roots, with the exception of fungi that show similar diversity between both biotopes of ‘SX64’. The general taxonomic structures of each microbial community were found to be cultivar- and biotope-specific. Although our universal fungal primers had the potential to amplify Glomeromycotina DNA, no sequences were assigned to this subphylum in all our samples, reinforcing the scientific idea that *Salix* spp. are mainly associated with EMF fungi. Among the fungi identified in our study, *Tuber borchii, Tomentella ellisii*, *Hymenogaster griseus, Geopora* spp., and *Hebeloma* spp., were found to be among the most dominant EMF partners of both cultivars. Some Dothideomycetes, Leotiomycetes, and Sordariomycetes were found to be abundant in the roots of both cultivars, suggesting that they are important endophytes for the health and growth of willows that grow under contaminated conditions. Our study is one of the few to provide information regarding archaea inhabiting the root zone of willows. It can be concluded that our two cultivars do not harbor endophytic archaea, because no sequence was obtained from PCR performed with all root samples. However, the high dominance of Thaumarchaeota in the rhizosphere suggested that most archaea associated with *S. miyabeana* are involved in nutrient cycling as ammonia oxidizers. This study gave a unique view into the root and rhizosphere microbiomes of *S. miyabeana* well established in a mixed contaminated soil for six years. Our findings and observations provide valuable and useful clues to a better understanding of plant- microbiome interaction, which could be used to improve agronomic techniques that rely on the use of microorganisms (inoculum and engineer) to increase the performances of willows under contaminated soil.

## Figures and Tables

**Table 1:** Soil characteristics of the site in 2010.

Parameters	Units	Values	Parameters	Units	Values
Cation-exchange capacity	meq 100 g^−1^	43.50	PCBs ^c^	mg kg^−1^	57.58 ± 11.70
pH ^a^	-	7.70	Cadmium ^c^	mg kg^−1^	1.75 ± 0.15
pH buffer	-	>7.50	Chromium ^c^	mg kg^−1^	659.50 ± 127.22
Soil texture	-	Clay	Copper ^c^	mg kg^−1^	1380.00 ± 201.57
Clay	%	46.00	Nickel ^c^	mg kg^−1^	42.90 ± 2.22
Silt	%	33.90	Lead ^c^	mg kg^−1^	34.00 ± 8.12
Sand	%	20.10	Zinc ^c^	mg kg^−1^	386.50 ± 72.13
Organic matter	%	9.60	Acenaphthene ^c^	mg kg^−1^	0.56 ± 0.18
K + Mg + Ca saturation	%	100.00	Acenaphtylene ^c^	mg kg^−1^	1.98 ± 0.38
P (P/Al) saturation	%	16.50	Anthracene ^c^	mg kg^−1^	18.15 ± 4.90
Ca saturation	%	81.60	Benz[a]anthracene ^c^	mg kg^−1^	0.43 ± 0.09
K saturation	%	3.10	Benzo[a]pyrene ^c^	mg kg^−1^	0.28 ± 0.07
Mg saturation	%	15.30	Benzo[ghi]perylene ^c^	mg kg^−1^	0.48 ± 0.12
**Parameters**	**Units**	**Values**	Chrysene ^c^	mg kg^−1^	0.40 ± 0.09
Al ^b^	mg kg^−1^	48.00	Fluoranthene ^c^	mg kg^−1^	0.54 ± 0.20
B ^b^	mg kg^−1^	1.40	Fluorene ^c^	mg kg^−1^	0.94 ± 0.21
Ca ^b^	mg kg^−1^	7090.00	Indeno [1,2,3-cd]pyrene ^c^	mg kg^−1^	0.32 ± 0.09
Cu ^b^	mg kg^−1^	417.00	Naphthalene ^c^	mg kg^−1^	0.42 ± 0.13
Fe ^b^	mg kg^−1^	178.00	Phenanthrene ^c^	mg kg^−1^	2.62 ± 0.71
K ^b^	mg kg^−1^	525.00	Pyrene ^c^	mg kg^−1^	1.34 ± 0.41
Mg ^b^	mg kg^−1^	800.00	1-Methylnaphthalene ^c^	mg kg^−1^	0.42 ± 0.13
Mn ^b^	mg kg^−1^	11.00	2-Methylnaphthalene ^c^	mg kg^−1^	0.42 ± 0.12
P ^b^	mg kg^−1^	80.00	1,3-Dimethylnaphthalene ^c^	mg kg^−1^	0.55 ± 0.18
Zn ^b^	mg kg^−1^	85.60	2,3,5-Trimethylnaphthalene ^c^	mg kg^−1^	0.40 ± 0.13

^a^ Water extraction. ^b^ Melich III method. ^c^ Chemical analysis was performed by AGAT Laboratories Ltd. (Montreal, QC, Canada) following the recommended provincial methods for environmental analyses [45,46,47,48,49]. Five soil samples were collected at 0–30 cm below ground in each plot (P1, P2, P3, and P4, see Figure 1A). Values are averages (mean ± SD, *n* = 20). The table was adapted from Guidi et al. [43].

**Table 2:** Shannon diversity index calculated on ASVs.

	SX61		SX64		*p*-Value			Interpretation
	Roots	Rhizosphere	Roots	Rhizosphere	Cultivar	Compartment	Cultivar × break//Compartment	
Fungi	1.95 ± 0.84	2.87 ± 0.63	2.52 ± 0.75	2.67 ± 0.89	0.385	**0.016**	**0.035**	**Roots.SX61 < Rhizo.SX61**
Bacteria	5.75 ± 0.31	6.92 ± 0.17	6.07 ± 0.37	7.07 ± 0.11	0.117	**<0.001**	**0.040**	**Roots.SX61 < Rhizo.SX61** **Roots.SX64 < Rhizo.SX64**
Archaea	-	0.68 ± 0.07	-	1.15 ± 0.59	0.158	-	-	-

Values are the averages (mean ± SD, *n* = 15) of the Shannon diversity index calculated on ASVs. Significance levels (*p*-value) are shown to indicate a significant difference between group samples. Bold indicates a significant difference between group samples.

**Table 3:** PERMANOVA analysis of the effects of the cultivar, plant compartment, and their interaction on fungal community structure, based on Euclidean distance.

Factor	Fungi				Bacteria				Archaea			
	Df	*F*.Model	R^2^	Pr (>F)	Df	*F*.Model	R^2^	Pr (>F)	Df	*F*.Model	R^2^	Pr (>F)
Cultivar	1	2.7657	0.0363	**0.006**	1	4.1311	0.0489	**0.002**	1	6.0091	0.1767	**0.001**
Compartment	1	16.3440	0.2145	**0.001**	1	23.0238	0.2725	**0.001**	-	-	-	-
Cultivar × Compartment	1	1.0759	0.0141	0.209	1	1.3507	0.0160	0.125	-	-	-	-
Residuals	56	-	0.7351	-	56	-	0.6627	-	28	-	0.8233	-
Total	59	-	1	-	59	-	1	-	29	-	1	-

Df, degree of freedom; *F*.Model, F-test value for model; R^2^, R-squared; Pr (>F), *p*-value. Bold indicates a significant effect of Cultivar, Compartment or Cultivar×Compartment on community structure.

## Data Availability

The datasets generated and analyzed during the current study are available in the Sequence Read Archive (SRA) under project number PRJNA803175. [https://www.ncbi.nlm.nih.gov/bioproject/803175] (accessed on 1 December 2021).

## Supplementary Materials

The following are available online at https://www.mdpi.com/article/10.3390/jof8020145/s1, Figure S1: Track visualization, Figure S2: Rarefaction curves, Figure S3: Krona charts of raw reads counts of all bacterial ASVs, Figure S4: Krona charts of raw reads counts of all archaeal ASVs, Figure S5: Boxplot of distance to centroid, Figure S6: MA-plots showing fold difference in the normalized count abundance ASVs, Figure S7: Most abundant ASVs showing significant differential abundances between two sample groups, Table S1: Tukey multiple comparisons of mean beta-dispersions for each group sample.
