# Supplementary material for: Ectomycorrhizal Fungi Dominated the Root and Rhizosphere Microbial Communities of Two Willow Cultivars Grown for Six-Years in a Mixed-Contaminated Environment
Source: J Fungi (Basel). 2022 Jan 30;8(2):145. doi: 10.3390/jof8020145 (PMC8880157; doi:10.3390/jof8020145)
Supplement: Supplementary file 1 [file jof-08-00145-s001.zip › jof-1541692-supplementary.pdf]

Journal of *Fungi*

MDPI

Supplementary Materials

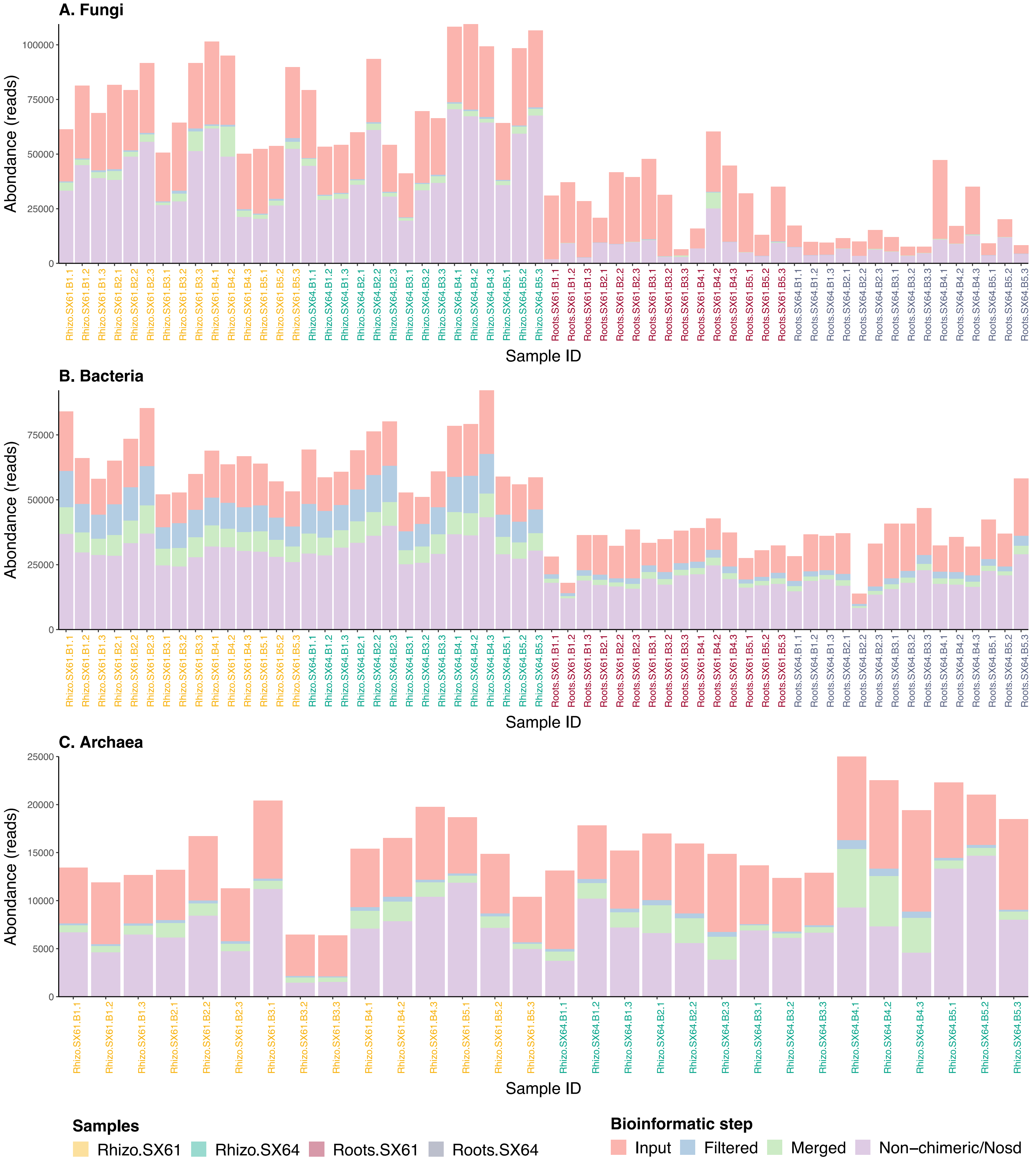

Figure S1. Track visualisation of (A) fungal; (B) bacterial; and (C) archaeal reads recovered from the whole dataset at different bioinformatic steps.

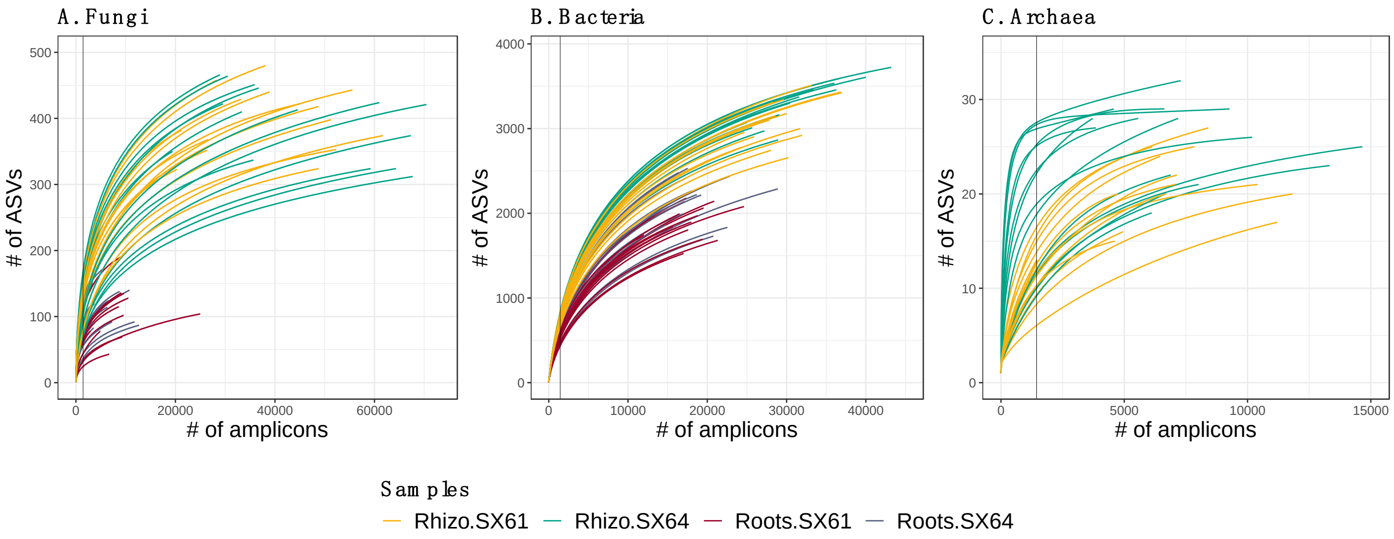

Figure S2. Rarefaction curves of (A) fungal; (B) bacterial; and (C) archaeal ASVs by sequence sample size.

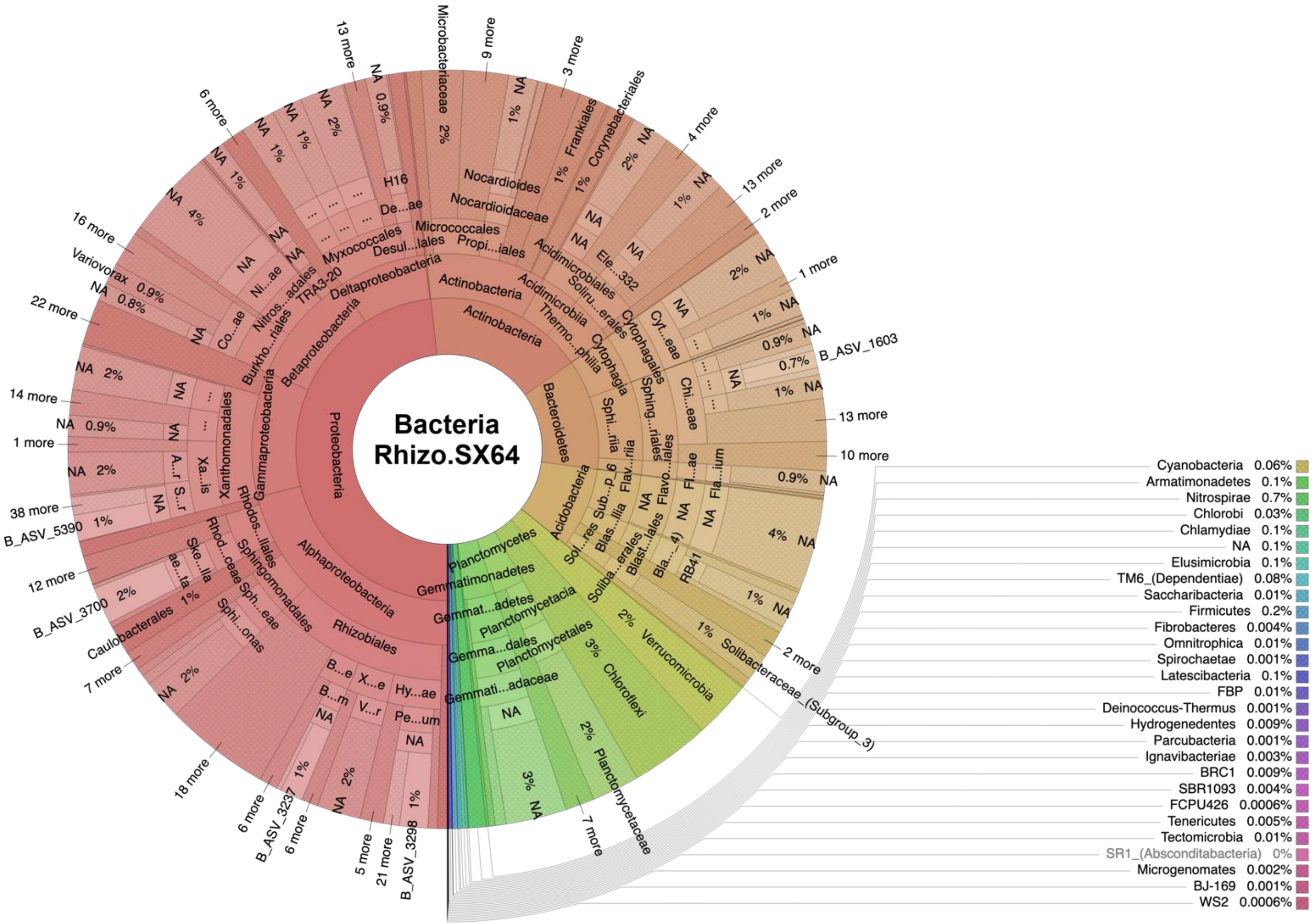

Figure S3. Krona charts of raw reads counts of all bacterial ASVs in each biotope of both *Salix* cultivars. Arc length are proportional to the relative number of reads by group (Rhizo.SX64 = 481,361 reads; Rhizo.SX61 = 448,274 reads; Roots.SX64 = 271,091 reads and Roots.SX61 = 271,933 reads). The interactive Krona charts are available at https://github.com/MaximeFortinFaubert/Figure3/blob/main/README.md.

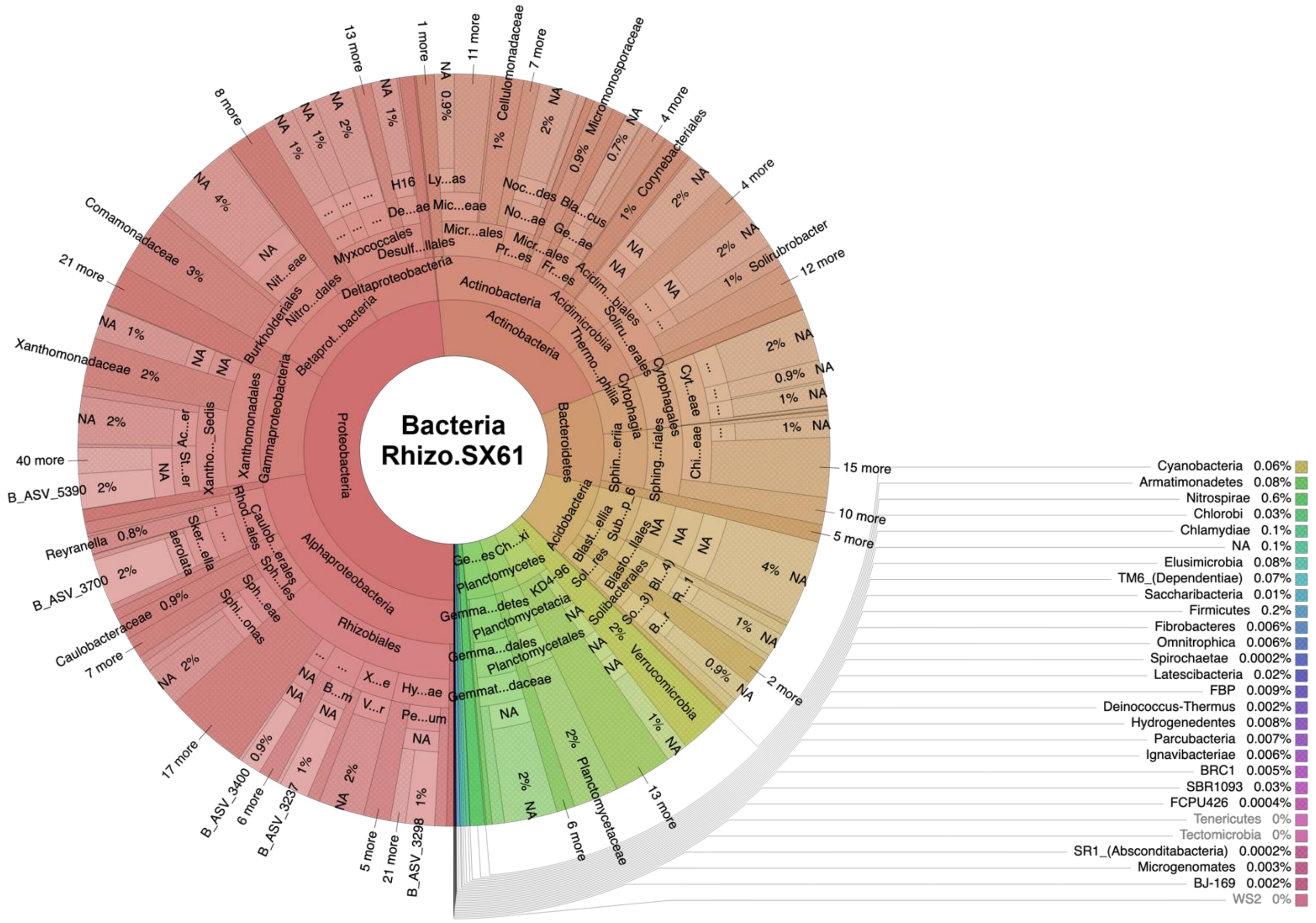

Figure S3. (continued)

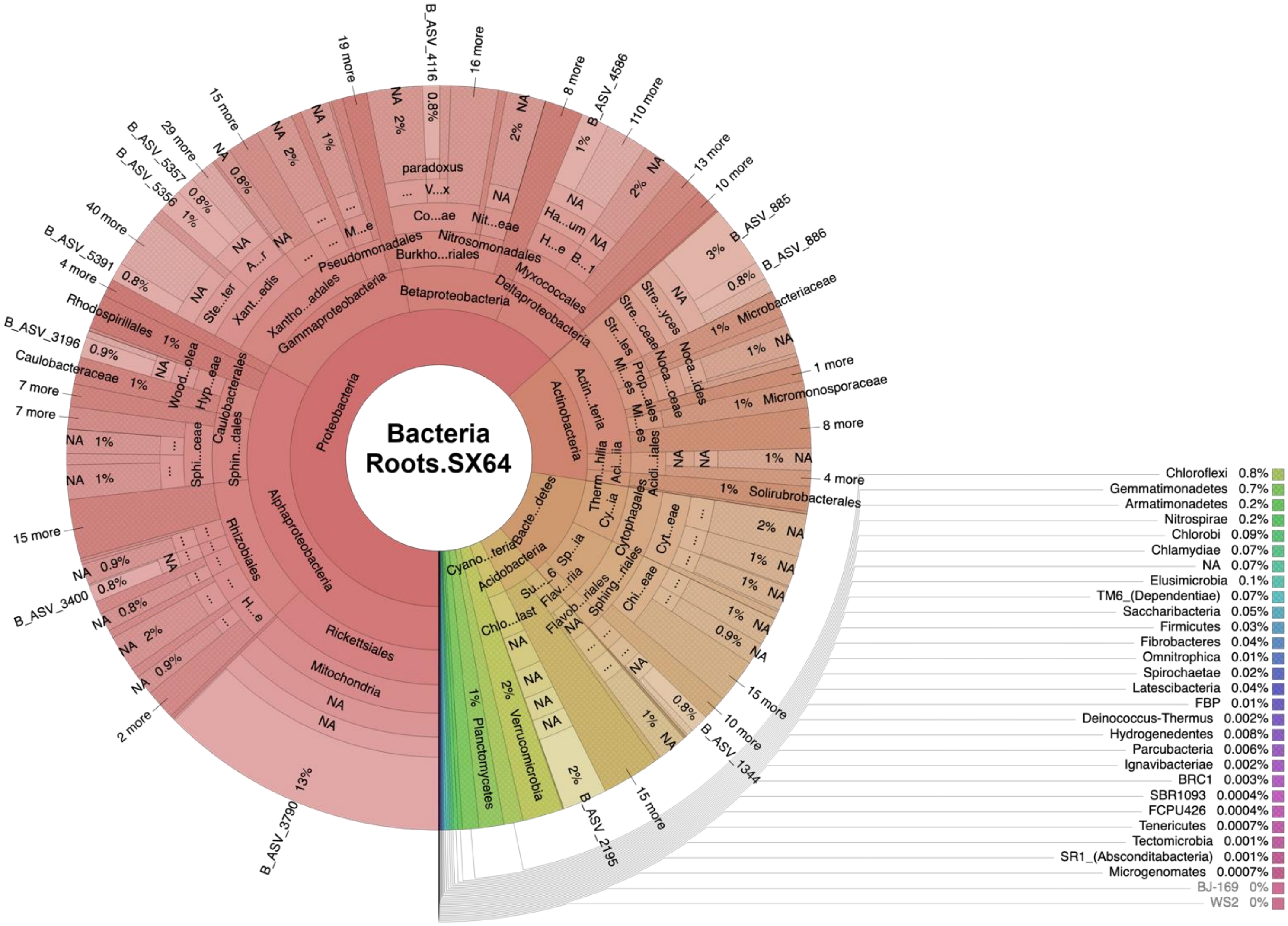

Figure S3. (continued)

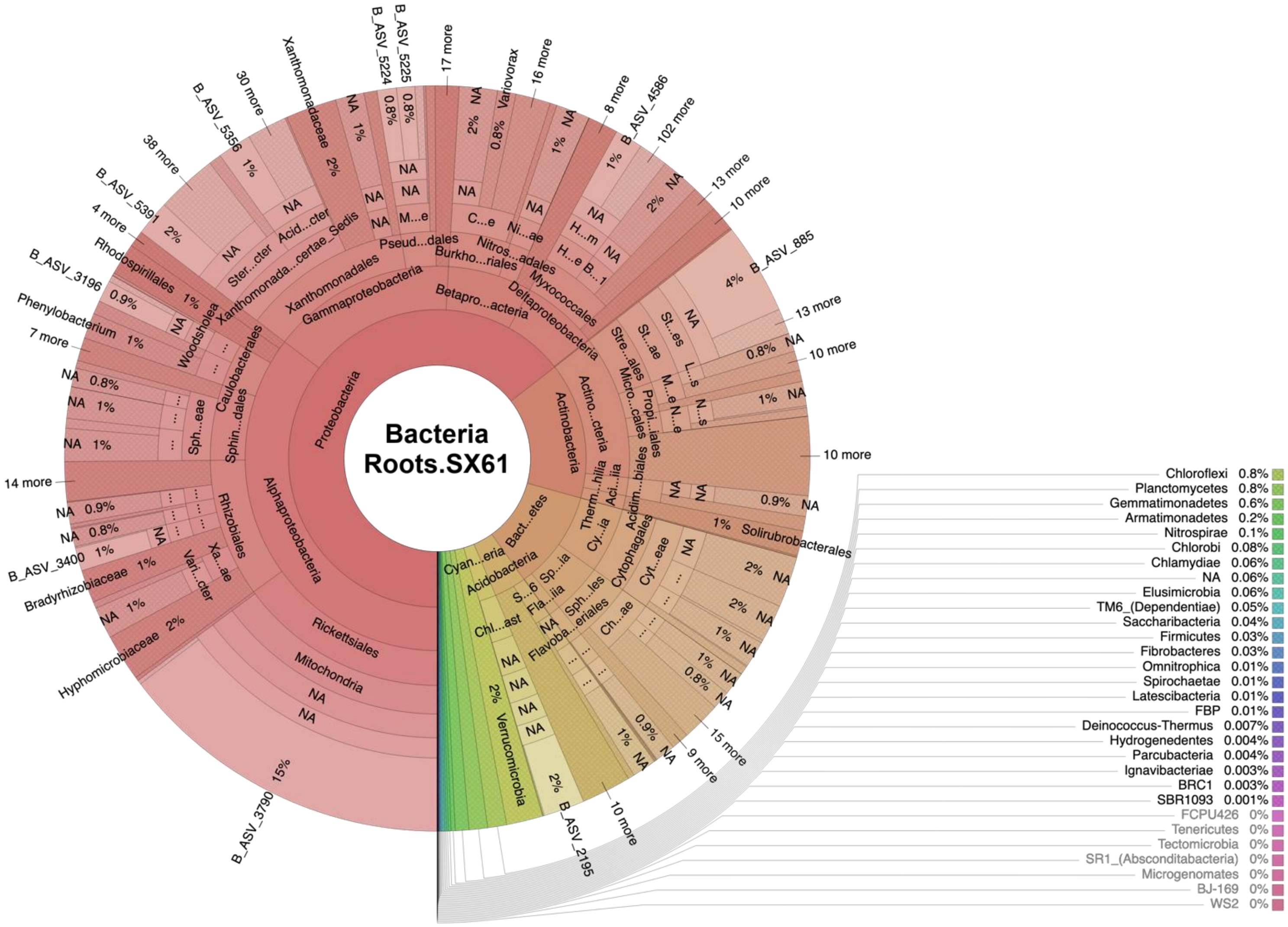

Figure S3. (continued)

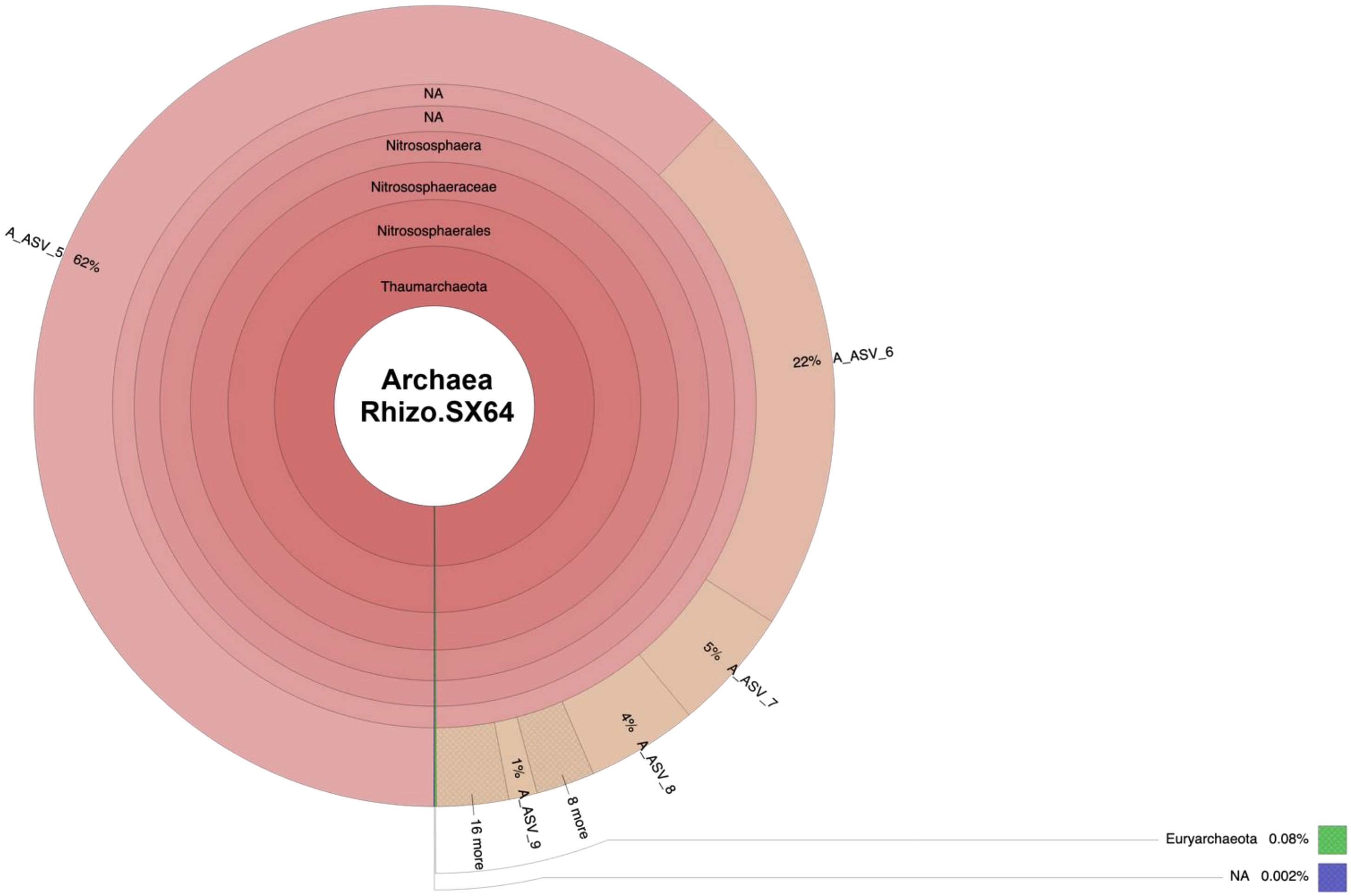

Figure S4. Krona charts of raw reads counts of all archaeal ASVs in each biotope of both Salix cultivars. Arc length are proportional to the relative number of reads by group (Rhizo.SX64 = 113,938 reads and Rhizo.SX61 = 100,483 reads). The interactive Krona charts are available at https://github.com/MaximeFortinFaubert/Figure4/blob/main/README.md.

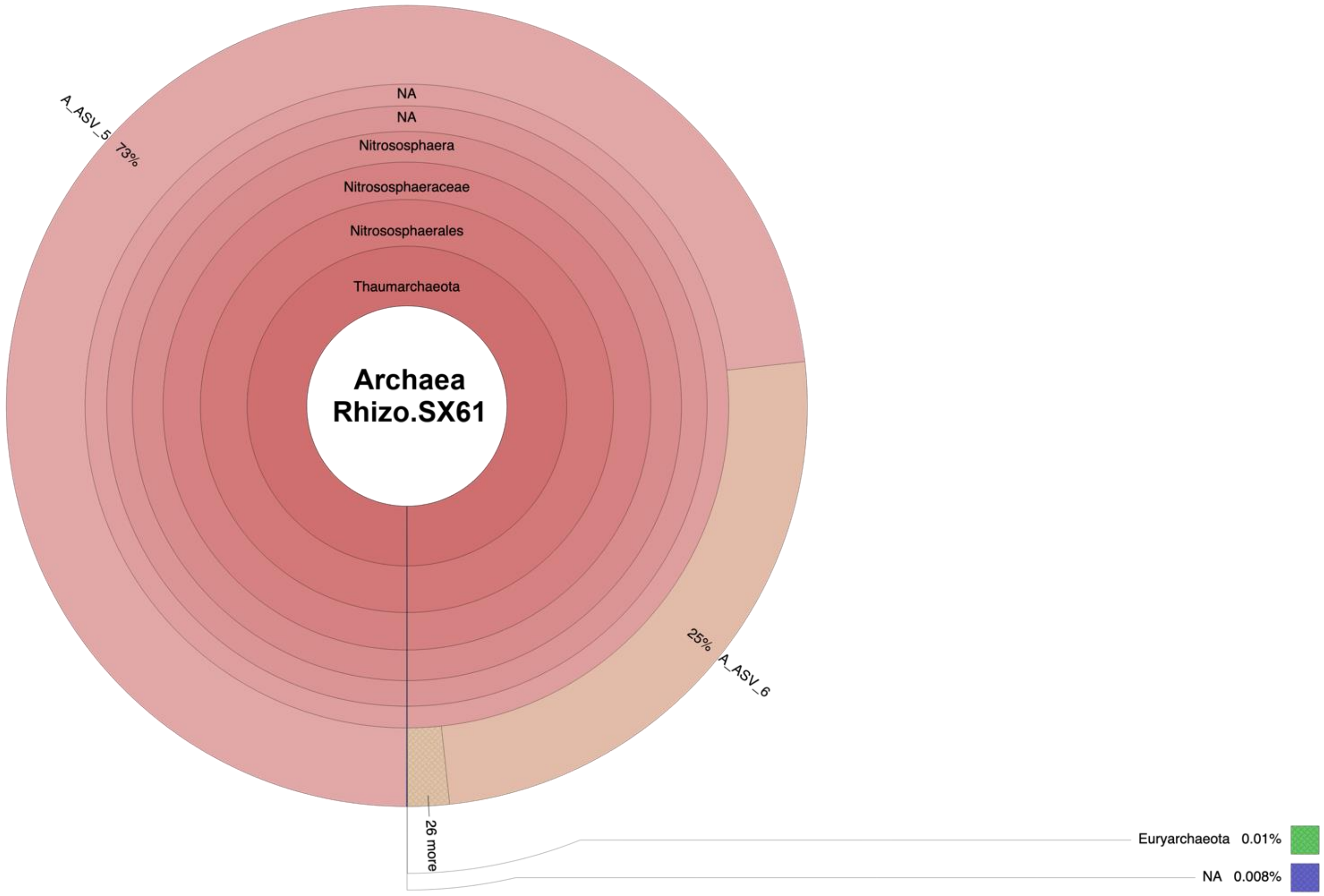

Figure S4. (continued)

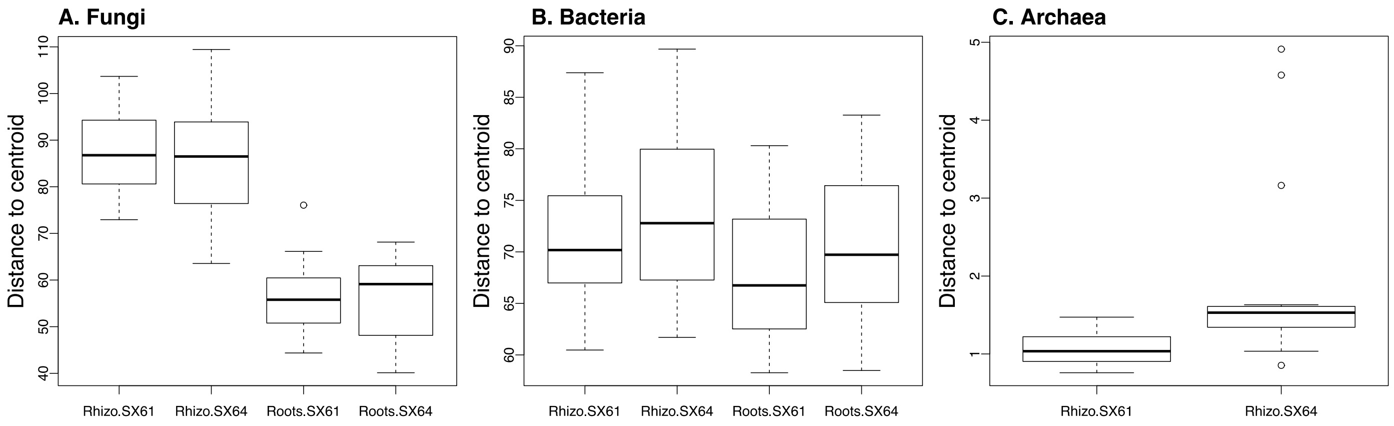

Figure S5. Boxplot of distance to centroid based on beta-dispersion analysis of (A) fungal; (B) bacterial; and (C) archaeal community in each group sample.

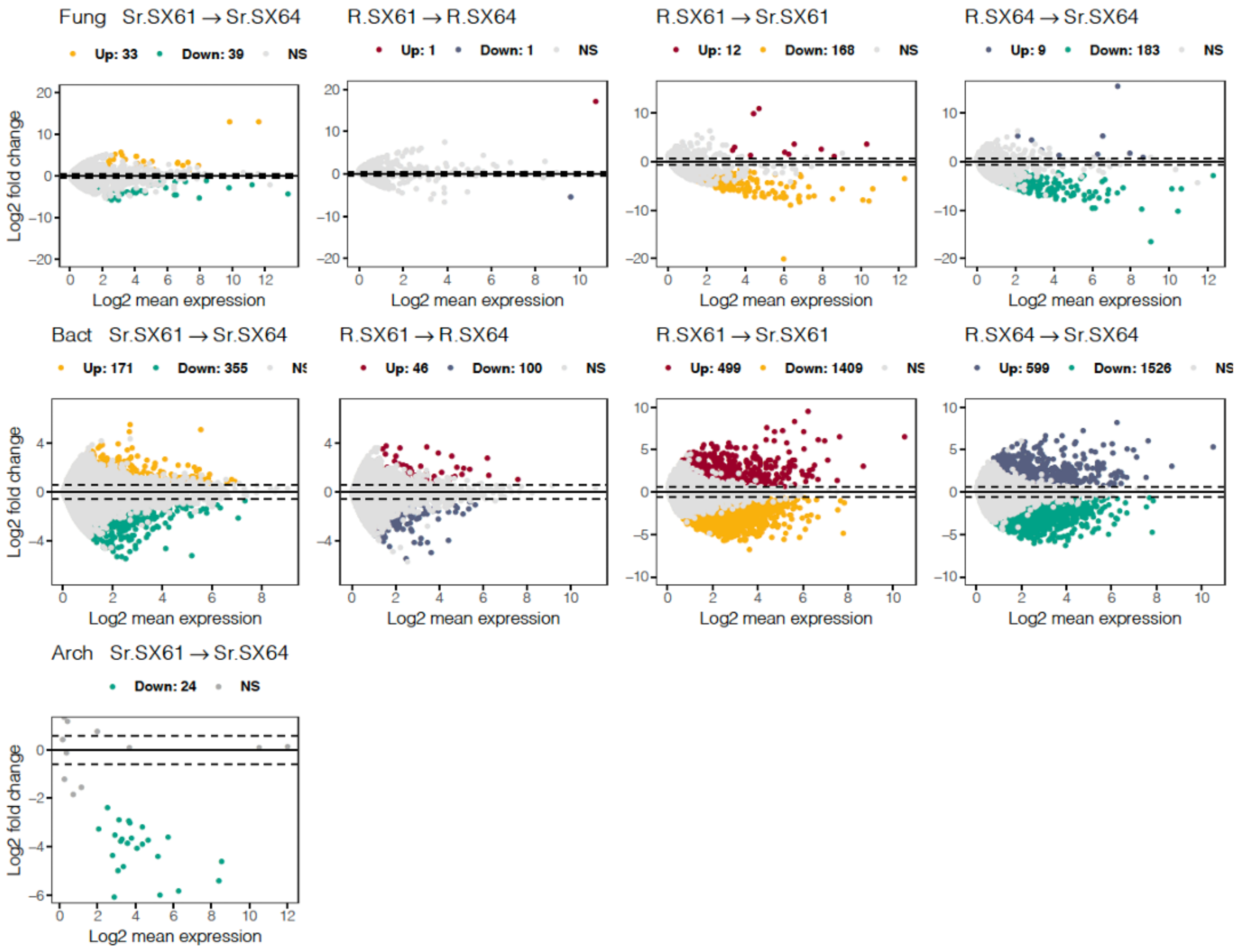

Figure S6. MA-plots showing fold difference in the normalized count abundance of ASVs between both cultivars and between their respective biotopes. All fungal (first line), bacterial (second line) and archaeal (third line) ASVs that showed significant differences between two groups (p_value_adj < 0.05) are highlighted in color according to the group hosting it higher normalized count abundance: Rhizo.SX61: Yellow; Rhizo.SX64: Green; Roots.SX61: Red and Roots.SX64: Blue. The gray dots are the other ASVs.

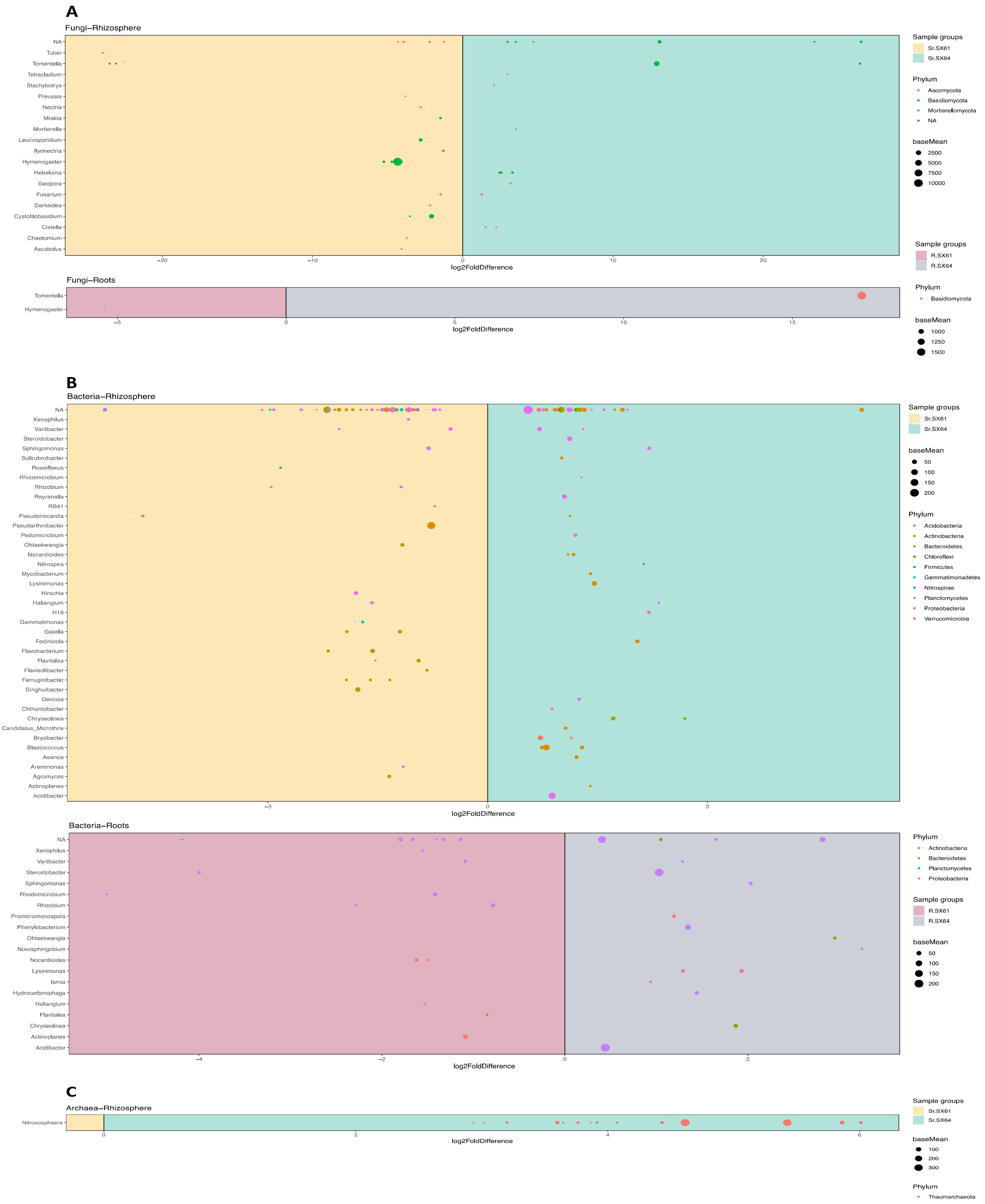

Figure S7. Most abundant (A) fugal; (B) bacterial, and (C) archaeal ASVs showing significant differential abundances between two sample groups. Dots indicate ASV, where their size are scaled by "baseMean" abundance and their color represent the phylum to which ASVs belongs. The background color of each ASV indicates in which sample group these ones are more abundant. Only ASVs with adjusted p-values < 0.05 and estimated base mean > 10 were considered significantly differentially abundant and included in these plots.

Table S1. Tukey multiple comparisons of mean beta-dispersions for each group sample.

Group sample	Interaction	Difference	Lower limit	Upper limit	p adj
Fungi	Rhizo.SX64 - Rhizo.SX61	-2.20859	-11.8735	7.456335	0.930
	Roots.SX61 - Rhizo.SX61	-31.1987	-40.8637	-21.5338	**<0.001*****
	Roots.SX64 - Rhizo.SX64	-29.2571	-38.922	-19.5921	**<0.001*****
	Roots.SX64 - Roots.SX61	-0.26691	-9.93183	9.398017	0.100
Bacteria	Rhizo.SX64 - Rhizo.SX61	2.063289	-5.12985	9.256429	0.872
	Roots.SX61 - Rhizo.SX61	-4.00992	-11.2031	3.183216	0.459
	Roots.SX64 - Rhizo.SX64	-2.86515	-10.0583	4.32799	0.718
	Roots.SX64 - Roots.SX61	3.208063	-3.98508	10.4012	0.641
Archaea	Rhizo.SX64 - Rhizo.SX61	0.871322	0.198514	1.54413	**0.013***

Significance levels (p-value) are shown and asterisks ($*p \leq 0.05$, $**p \leq 0.01$, $***p \leq 0.001$) indicate a significant difference between two group samples.
